# The leaching of phthalates from PVC can be determined with an infinite sink approach

**DOI:** 10.1016/j.mex.2019.10.026

**Published:** 2019-11-06

**Authors:** Charlotte Henkel, Thorsten Hüffer, Thilo Hofmann

**Affiliations:** a Research Platform Plastics in the Environment and Society –PLENTY, University of Vienna, Althanstrasse 14, 1090, Vienna, Austria; b Department of Environmental Geosciences, Centre for Microbiology and Environmental Systems Science, University of Vienna, Althanstrasse 14, 1090, Vienna, Austria

**Keywords:** PVC, polyvinyl chloride, DEHP, bis(2-ethylhexyl) phthalate, DEHP-d4, deuterated bis(2-ethylhexyl) phthalate, An infinite sink approach to investigate the leaching of phthalates from PVC, Release, Plasticiser, Microplastics, Aquatic environment, GC–MS

## Abstract

Polyvinyl chloride (PVC) is the third most used polymer for plastic products in the European Union (+NO/ CH) and contains the highest amounts of additives, especially phthalic acid esters (phthalates). Leaching kinetics of additives from (micro-) plastics into aqueous environments are highly relevant for environmental risk assessment and modelling of the fluxes of plastics and its associated additives. Investigating the leaching of phthalates into aqueous environments in batch experiments is challenging due to their low solubility and high hydrophobicity and there are no standard methods to study release processes. Here we describe an infinite sink method to investigate the leaching of phthalates from PVC into the aqueous phase. Spiking and leaching experiments using bis(2-ethylhexyl) phthalate as a model phthalate enabled the validation and evaluation of the designed infinite sink method. The developed method offers:

• a low-cost and simple approach to investigate leaching of phthalates from PVC into aqueous environments
• the use of a high-surface activated carbon powder as an infinite sink
• a tool to elucidate the transport fluxes of plastics and additives

## Graphical abstract

**Figure:**
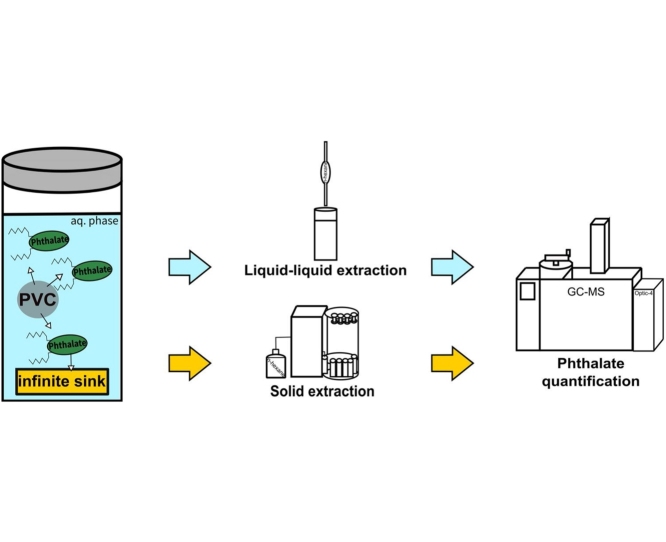

**Specification Table**

**Table:** 

Subject Area:	Environmental Science
More specific subject area:	N/A
Method name:	An infinite sink approach to investigate the leaching of phthalates from PVC
Name and reference of original method:	N/A
Resource availability:	N/A

## Method details

### Reagents and standards

N-hexane (≥95 %, PESTINORM®), acetone (Anala® NORMAPUR) and 2-propanol (Anala® NORMAPUR) were purchased as solvents from VWR Chemicals (Vienna, Austria). Ultra-pure water was obtained from a water purification system (Milli-Q gradient A 10, Milipore, Merck, Darmstadt, Germany). Norit® SAE SUPER (Cabot Norit Nederland B.V., Klazienaveen, The Netherlands) was used as activated carbon powder. Grade 50 filter paper was purchased from Whatman (GE Heathcare, Dassel, Germany). Copper wire (0.6 mm thickness) was from HSB Elektro (Uslar, Germany). DEHP **(**analytical standard) and DEHP-d4 (Pestanal®, analytical standard) were purchased from Merck **(**Darmstadt, Germany). DEHP and DEHP-d4 stock standards (each 5000 μg mL^−1^) were prepared in 2-propanol. From these working standards of DEHP (50 μg mL^−1^ and 500 μg mL^−1^) and DEHP-d4 (50 μg mL^−1^) were made in 2-propanol. All standards were stored at 4 °C in dark in amber brown glass vials closed with caps equipped with teflon septa. A 1 mM potassium chloride (KCl) solution was used as background solution to stabilise the ionic strength. PVC pellets were purchased from Industrie Generali spa (Samarate, Italy) and contained ∼35 % of DEHP. Each leaching or spiking experiment was conducted in triplicates, including three blanks.

### Preparatory

Glassware (20 mL and 60 mL vials) were cleaned with ultra-pure water, dried and rinsed with acetone. All glassware were heated at 550 °C for six hours in a muffle oven and closed with caps equipped with teflon septa.

The infinite sink consisted of 10 mg activated carbon powder packed in a 4 cm x 4 cm piece of Grade 50 filter paper. A copper wire kept the rectangular infinite sink in shape and increased the mechanical stability. A comparison with other filter papers and the decision for the Grade 50 filter paper are discussed in the *Supplementary Material*. The infinite sink was added to a 60 mL glass vial filled with 40 mL of the 1 mM KCl solution. The vial with the infinite sink was placed on a horizontal shaker (in dark to prevent photodegradation) at room temperature and 125 rpm for a minimum of 12 h to equilibrate the sink with the KCl solution.

**Fig. 1:**
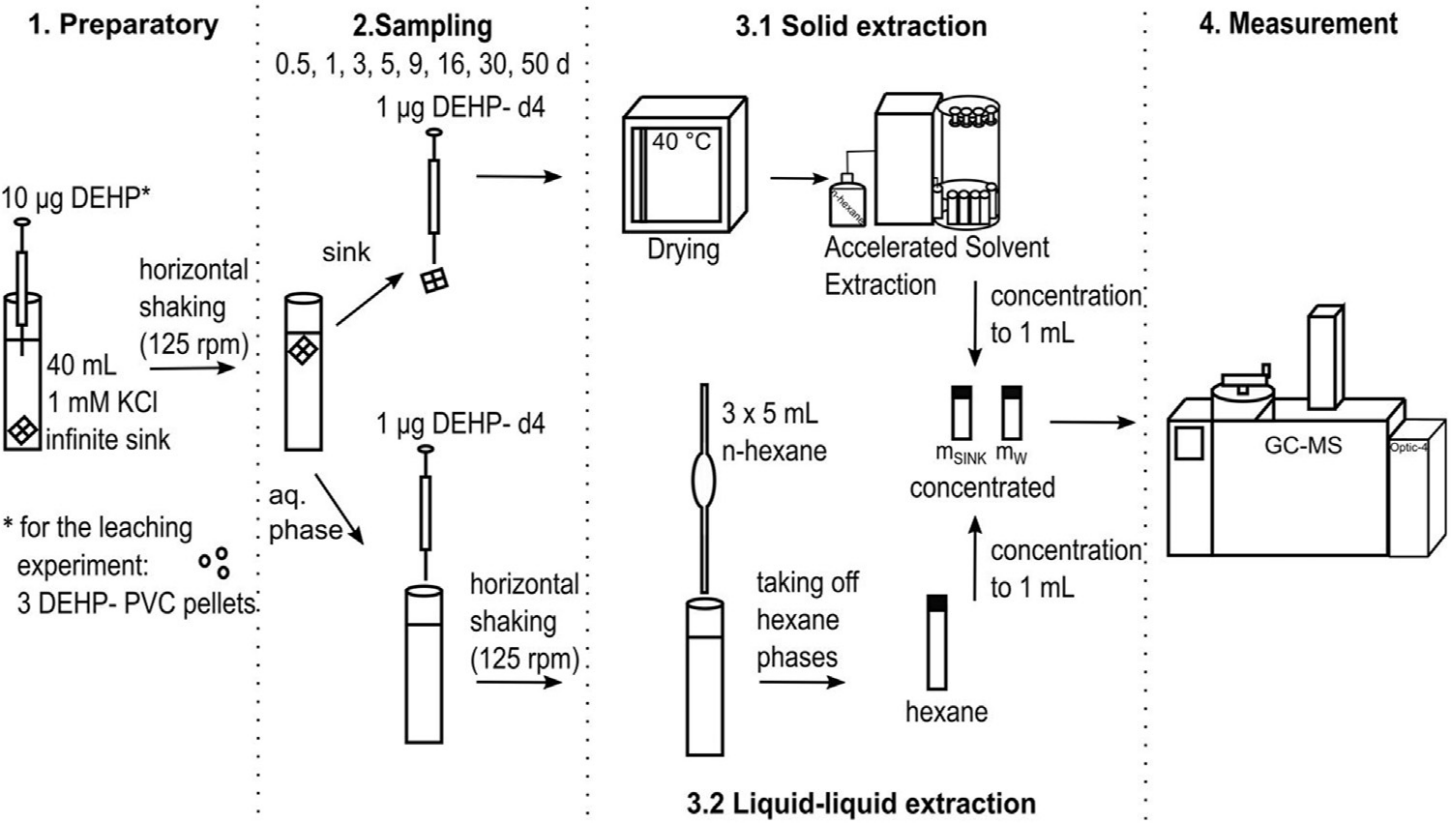
Experimental procedure of the infinite sink method.

An infinite sink should take up phthalates from the aqueous phase and keep their concentration well below the solubility. A spiking experiment provided the information on the capacity and velocity of the infinite sink to remove phthalates from the aqueous phase. 20 μL of a 500 μg mL^−1^ DEHP standard were spiked into the KCl solution containing the infinite sink. For the leaching experiment, three PVC pellets (∼ 85 mg) were added to each vial. The pellets should have a similar size, weight and shape and thus a similar surface area. The vials with the pellets were horizontally shaken at 125 rpm and room temperature until sampling. To determine the method blank value, samples containing only the background solution and the infinite sink were prepared. A summary of the experimental protocol is provided in Fig. 1.

### Sampling

Due to the hydrophobicity of phthalates, the leaching experiment was conducted using sampling intervals of 0.5, 1, 3, 5, 9, 16, 30 and 50 days. The sampling intervals were adopted for the spiking experiment. At each sampling time, the infinite sinks were removed using tweezers. For the leaching experiment, first the PVC pellets and then the infinite sinks were removed. For the quantification of DEHP in the aqueous phase and in the infinite sink, both phases were spiked with 20 μL of a 50 μg mL^−1^ DEHP-d4 standard. The infinite sinks were dried at 40 °C oven temperature for a minimum of 12 h. The dried infinite sinks were stored in a desiccator until extraction. Following the spiking of the DEHP-d4 standard to the aqueous phases, the vials were horizontally shaken until extraction.

### Extraction

#### Solid phase extraction

The infinite sinks were extracted using Accelerated Solvent Extraction (ASE 200, Thermo Fisher, Waltham, US). The extraction was conducted in two cycles at 120 °C and 40 bar for seven minutes in 11 mL cylinder bodies using n-hexane as a solvent. The solid phase extraction for each sample resulted in ∼30 mL of n-hexane extract, which was transferred in portions of 15 mL into a 20 mL glass vial and concentrated before the refill.

#### Liquid-liquid extraction

To extract DEHP from the aqueous phase 5 mL of n-hexane were added. The vial containing both phases was vigorously shaken by hand for 2 min and placed in a vial rack. After the water and n-hexane phase separated, the n-hexane phase was transferred into a 20 mL glass vial. The extraction was repeated three times and the n-hexane extracts were pooled in the same 20 mL vial.

The n-hexane extracts were concentrated to 1 mL using a laboratory evaporator (Barkey vapotherm basic mobil I, Leopoldshöhe, Germany) at 40 °C and nitrogen aeration. The concentrated extracts were transferred into 1.5 mL brown glass measurement vials and stored at 4 °C until measurement.

### Measurement

The DEHP concentration was determined using a GC–MS system consisting of a 7980A gas chromatograph (Agilent Technologies, Santa Clara, US) equipped with an OPTIC-4 Multimode Inlet (GL Sciences B.V., Eindhoven, The Netherlands) and coupled to a 5975C single quadrupole mass spectrometer (Agilent Technologies, Santa Clara, US).

The detailed instrumental conditions are given in the *Supplementary Material.* To determine the response factor between DEHP and DEHP-d4 an external calibration was carried out using 0.5, 1, 3, 5, 10, 15 μg mL^−1^ of DEHP standards. These standards were prepared adding the appropriate amount of DEHP standard (50 μg mL^-1^ or 500 μg mL^−1^), n-hexane and 20 μL of DEHP-d4 (50 μg mL^−1^) to a 1.5 mL brown glass measurement vial. The linear regression of the calibration curve yielded a correlation coefficient of R² > 0.999. The limit of detection (LOD) and the limit of quantification (LOQ) were determined using signal-to-noise (S/N) ratios of 3:1 and 10:1, respectively. A LOD of 0.03 μg mL^−1^ DEHP and a LOQ of 0.25 μg mL^−1^ DEHP were found. Method blanks were below the detection limit. To ensure stable conditions hydrochemical parameters were observed throughout the experiment and are given in the *Supplementary Material*.

### Method validation and data evaluation

**Fig. 2:**
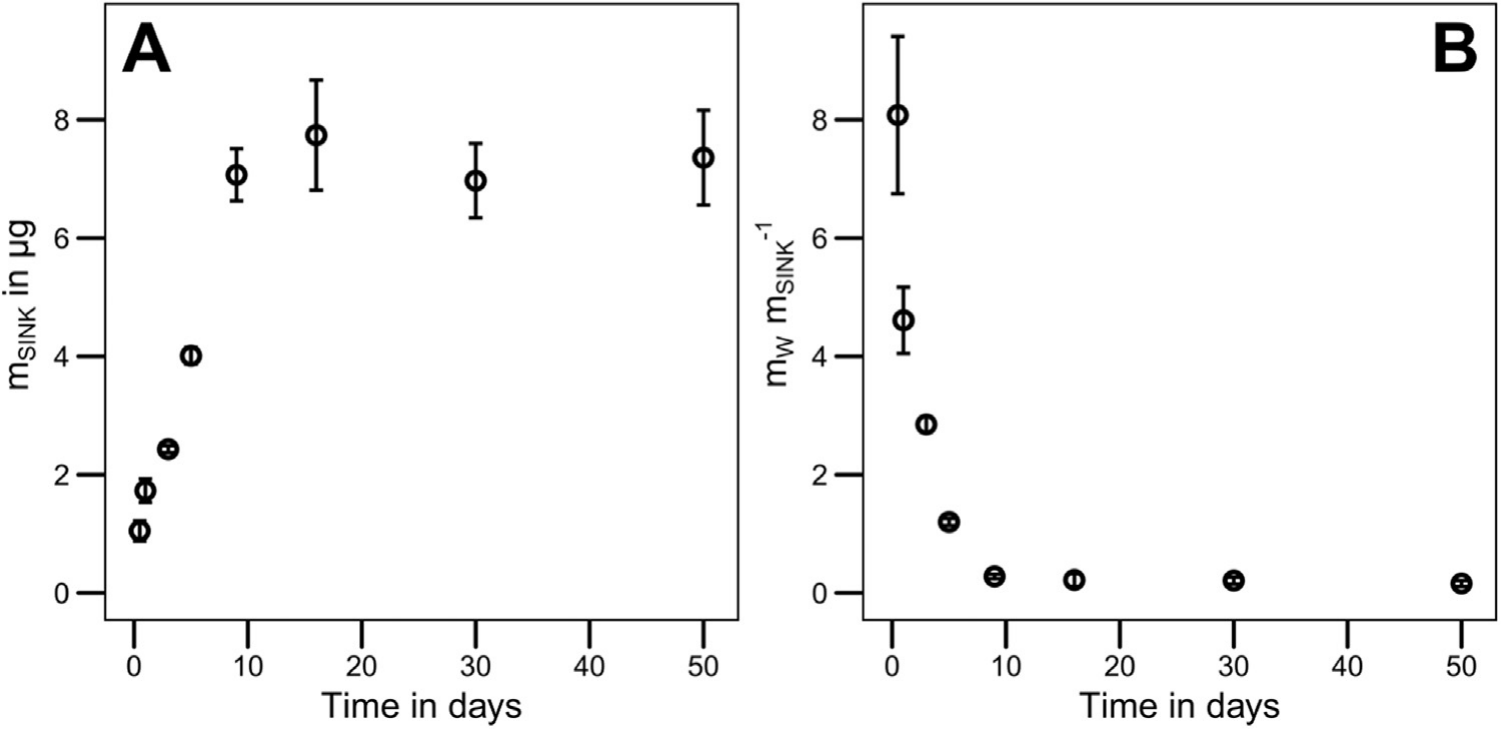
Data evaluation of the spiking experiment (error bars indicate the standard deviation, n = 3). Fig. 2A shows the mass of DEHP in the infinite sink (m_SINK_) in μg at each sampling time. Fig. 2B illustrates the ratio of DEHP mass in the aqueous phase and in the infinite sink (m_W_ m_SINK_^−1^) at each sampling time.

The experimental procedure provided: the mass of DEHP in the infinite sink (m_SINK_) and in the aqueous phase (m_W_) for each sample. For the spiking experiment, the mass balance (m_TOT_= m_SINK_ + m_W_) and the recovery of DEHP were calculated. The recovery of DEHP ranged between 85 % and 98 %. For the spiking experiment, the equilibrium of DEHP sorbed to the infinite sink was reached after nine days (Fig. 2A). A decreasing ratio of m_W_ m_SINK_^−1^ over time indicated that the infinite sink strongly removed DEHP from the aqueous phase during the spiking experiment and kept the DEHP concentration well below the solubility (Fig. 2B), as desired.

**Fig. 3:**
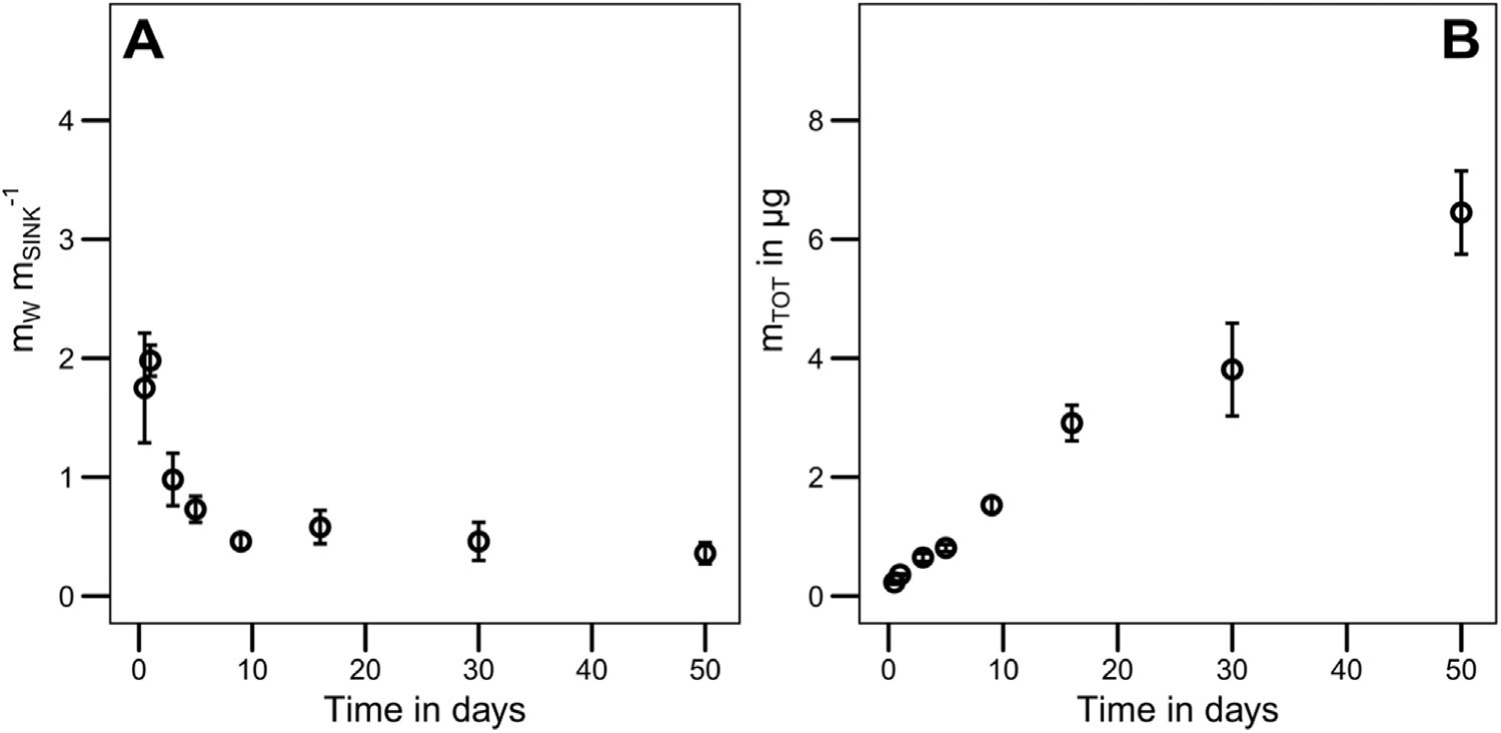
Data evaluation of the leaching experiment (error bars indicate the standard deviation, n = 3). Fig. 3A illustrates the ratio of DEHP mass in the aqueous phase and in the infinite sink (m_W_ m_SINK_^−1^) at each sampling time. Fig. 3B shows the total mass (m_TOT_) of DEHP leached from the PVC pellets in μg at each sampling time.

For the leaching experiment the ratio m_W_ m_SINK_^−1^ likewise decreased as the infinite sink effectively removed DEHP from the aqueous phase (Fig. 3A). It was possible to demonstrate a time-dependent leaching curve of the total amount of DEHP m_TOT_ released from PVC pellets (Fig. 3B). The presented infinite sink method describes a novel approach to investigate the leaching behaviour and release kinetics of phthalates from PVC to the aquatic environment.

## Additional information

PVC is the third most often used polymer for many plastic products, ranging from medical equipment to toys for children, furniture and industrial pipelines [1,2]. Amongst the most widely used polymers, PVC contains the highest amounts of additives, with typically about 30% – 35% of phthalates like DEHP [3,4]. The additives are not chemically bond to the polymer and can transfer to the surrounding media like air, soil and water within the lifespan of the plastic products, which also changes the mechanical properties of the plastics [5,4]. Some phthalates like DEHP and diethyl phthalate are toxic, endocrine disrupting chemicals and on the US EPA list of priority pollutants [2,6,7]. Leaching kinetics of additives from (micro-) plastics pose one of the main research gaps [8]. Understanding leaching processes of phthalates from PVC is therefore highly relevant for an environmental risk assessment and modelling of transport fluxes of plastics and its associated additives. The low solubility of phthalates (e.g. DEHP ∼ 270 μg L^−1^) and their high partitioning coefficients (log K_OW, DEHP_ = 7.6) hamper the investigation of the leaching of phthalates from PVC using a two-phase experimental set-up including the plastics and an aqueous phase [9]. The introduction of a third phase, which operates as an ‘infinite sink’ for the leaching substance offers a promising approach. A sufficiently large surface area of the sink material and affinity for the solute are required to ensure the kinetics are governed by the desorption of the solute to the leachate and not by the sorptive uptake of the sink [10]. ‘Infinite sinks’ were used to investigate the desorption of polycyclic aromatic hydrocarbons from contaminated soil samples and to determine the release kinetics of polychlorinated biphenyls from polyethylene pellets [11,10]. Approaches to investigate leaching of phthalates from PVC are limited and present methods focus on leaching of phthalates into organic solvents or into water at elevated temperatures [4,6]. The infinite sink approach presented here used a high-surface activated carbon powder to investigate the leaching of phthalates from PVC into the aqueous phase.
