# Supplementary material for: The leaching of phthalates from PVC can be determined with an infinite sink approach
Source: MethodsX. 2019 Nov 6;6:2729–34. doi: 10.1016/j.mex.2019.10.026 (PMC6880001; doi:10.1016/j.mex.2019.10.026)
Supplement: Supplementary file 1 [file mmc1.docx]

**Supplementary Material**

**Comparison and evaluation of different filter papers:**

To develop the presented infinite sink approach, the selection of a filter paper is crucial. The selected filter paper has to meet the following requirements:

1. Highly permeable for water and non-permeable for the infinite sink material (activated carbon powder).

2. Stable during the preparation and the duration of the leaching experiment.

3. Polymer-free as during the solid extraction of the infinite sink using Accelerated Solvent Extraction (ASE), polymers extracted from the filter paper may lead to clogging of the valve.

4. Phthalate-free as low blanks are required.

5. Free from any organic compounds, which have a similar mass and retention time in the GC-MS chromatogram as the compound of interest.

Considering these criteria, five filter papers differing in their thickness and material composition were investigated. An overview of the filter papers under investigation is given in Table S1. A comparison of the stability of the infinite sinks prepared using these filter papers is provided in Fig. S1.

Table S1: Materials and thicknesses of the investigated filter papers.

| **Filter paper** | **Material** | **Thickness (mm)** |
| --- | --- | --- |
| **Grade 50**  (Whatman, GE Heathcare, Dassel, Germany) | paper | 0.115 |
| **Blue Ribbon 589/3**  (Whatman, GE Heathcare, Dassel, Germany) | paper | 0.16 |
| **Grey pack 640 w**  (Macherey Nagel, Düren, Germany) | paper | 0.20 |
| **Black Ribbon 589/1**  (Whatman, GE Heathcare, Dassel, Germany) | paper | 0.19 |
| **GF/ A**  (Whatman, GE Heathcare, Dassel, Germany) | glass fibres | 0.26 |


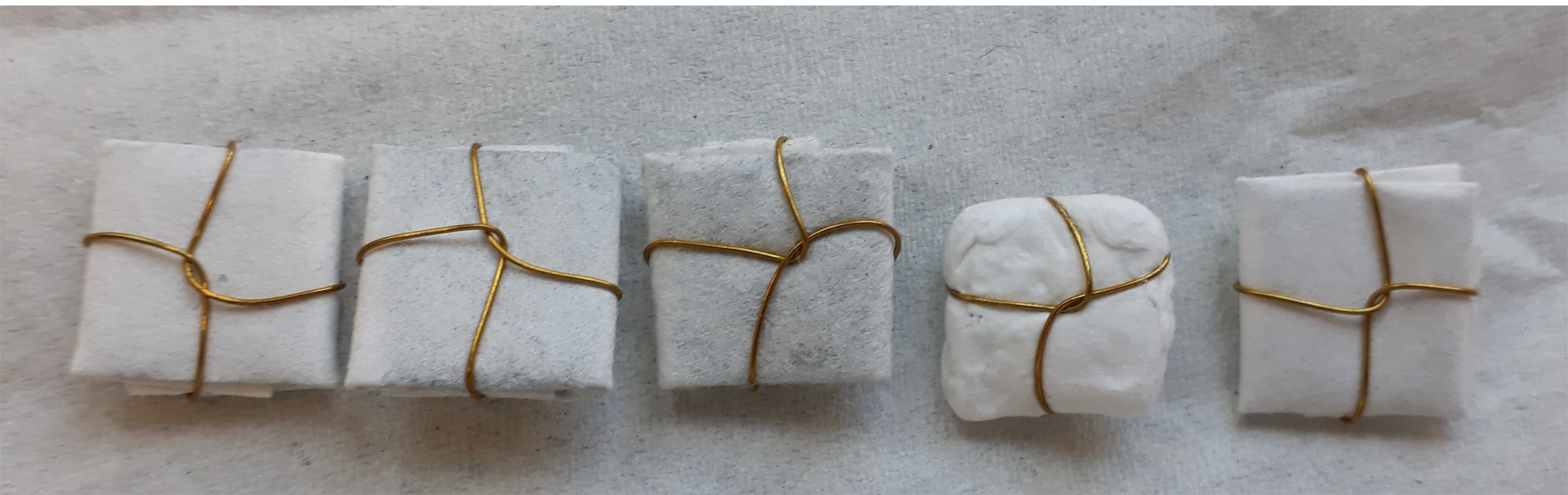


Fig. S1: Comparison of the stability of the different filter papers for an infinite sink approach after their application for spiking experiments. From left to right: Blue Ribbon 589/3, Black Ribbon 589/1, Grey Pack 640 w, GF/ A, Grade 50.

**Grade 50:**

Grade 50 filter paper showed the best performance during spiking and leaching experiments. This filter paper stood out due to a high stability during the preparation and the application of the infinite sink (Fig. S1). Infinite sinks made using this filter paper contained no detectable amounts of phthalates or any organic compounds overlapping with the phthalate peaks in the GC-MS chromatograms. Therefore, Grade 50 filter paper was most suitable for the infinite sink approach using activated carbon powder.

**Blue ribbon 589/3:**

Blue Ribbon 589/3 filter paper showed good performance during spiking and leaching experiments. The infinite sinks prepared using this filter paper contained no detectable amount of phthalates or any organic compounds overlapping with the phthalate peaks in the GC-MS chromatograms. However, the stability was lower compared with those of the Grade 50 filters (Fig. S1).

**Grey Pack 640 w and Black Ribbon 589/1:**

Grey Pack 640 w and Black Ribbon 589/1 filter papers showed poor suitability for the infinite sink approach. During the spiking experiments, the infinite sinks prepared using these filter papers started to disintegrate. Activated carbon powder and fibres separated from the filter papers were found in the aqueous phase and on the surface of the infinite sinks packages (Fig. S1). Using the Grey pack 640 w and Black Ribbon 589/1 filter papers resulted in blanks of 1.19 +- 0.0504 µg and 0.463 +- 0.147 µg DEHP per infinite sink package, respectively, which was too high to investigate the leaching of DEHP from PVC. Pre-cleaning of both filter papers with n-hexane reduced their stability. Due to the poor performance of both filter papers during the spiking experiments, they were not considered for leaching experiments using DEHP- containing PVC pellets.

**GF/ A:**

GF/ A filter papers also showed poor suitability. Infinite sinks packages composed of this filter paper contained no detectable amounts of phthalates. Since the infinite sink packages using this filter material deformed and partly disintegrated during the spiking experiment (Fig. S1), it was not considered for leaching experiments.

GC-MS conditions for the analysis of phthalates

Table S2: GC-MS conditions for the analysis of phthalates.

| **Gaschromatograph**  Column  Dimensions  Film thickness  Carrier gas  Temperature program | 7980A (Agilent Technologies, Santa Clara, US)  HP- 5MS (Agilent J&W, Santa Clara, US)  60 m x 0.25 mm  0.25 µm  Helium 5.0 (Linde Gas GmbH, Stadl-Paura, Austria)  80 °C (0.5 min)  20 °C/min to 300 °C (10 min) |
| --- | --- |
| **Injector**  Injection  Inlet temperature  Initial temperature  Ramp rate  Final temperature  Hold time  Column flow/ Inlet pressure  Transfer column flow  Transfer time/ splitless time  Column flow  Split flow | OPTIC-4 (GL Sciences B.V., Eindhoven, The Netherlands)  1 µL splitless  55 °C  10 °C/sec  350 °C  600 sec  2.6 mL/min  60 sec  1 mL/min  20 mL/min |
| **Mass spectrometer**  Source temperature  Quad temperature  Solvent delay  Acquisition mode  For DEHP  Ions | 5975C (Agilent Technologies, Santa Clara, US)  230 °C  150 °C  8 min  Selected ion monitoring  Mass: 149, 153, 167  171, 207, 279  283, 293 |

**Hydrochemical conditions during the spiking and the leaching experiments**

The concentrations of total organic carbon (TOC), copper and the pH value were determined for each sampling time (Table S3). The TOC concentration was measured using a Total Organic Carbon Analyser (TOC- LCPH/CPN, Shimadzu, Kyoto, Japan), the copper was determined by an Agilent 7900 quadrupole ICP-MS (Agilent Technologies, Santa Clara, US), pH was measured using a Multi 9620 IDS multiparameter benchtop meter (WTW, Weilheim, Germany).

Table S3: Hydrochemical parameters determined throughout the experiment. Given are the mean (n= 3) and the standard deviation of the total organic carbon (TOC) and copper concentrations and of the pH values for each sampling time.

| **Sampling time in days/ parameter** | **0.5** | **1** | **3** | **5** | **9** | **16** | **30** |
| --- | --- | --- | --- | --- | --- | --- | --- |
| **TOC in mg L^-1^** | 0.84 ± 0.06 | 1.08 ± 0.11 | 1.16 ± 0.03 | 1.21 ± 0.06 | 0.89 ± 0.10 | 1.20 ± 0.19 | 1.20 ± 0.15 |
| **Copper conc. in ppb** | 10.6 ± 2.0 | 18.4 ± 4.6 | 25.1 ± 3.1 | 42.5 ± 7.0 | 36.3 ± 7.5 | 148 ± 29 | 298 ± 48 |
| **pH** | 7.07 ± 0.03 | 7.11 ± 0.06 | 7.27 ± 0.04 | 7.25 ± 0.03 | 7.15 ± 0.09 | 7.14 ± 0.03 | 7.14 ± 0.02 |
